# Factors associated with the 6-minute walk test performance in older adults with hyperkyphosis

**DOI:** 10.1016/j.gerinurse.2022.07.003

**Published:** 2022-07-25

**Authors:** Yoshimi Fukuoka, Wendy B. Katzman, Amy Gladin, Nancy E. Lane, Jung Oh Yoo

**Affiliations:** a Department of Physiological Nursing, University of California, San Francisco, CA, USA; b Department of Physical Therapy and Rehabilitation Science, University of California, San Francisco, CA, USA; c Department of Pain Medicine, Kaiser Permanente Northern California, San Francisco Medical Center, San Francisco, CA, USA; d Department of Medicine, Division of Rheumatology and Immunology, School of Medicine, University of California, Davis, CA, USA; e Department of Communication, University of California, Davis, CA, USA

**Keywords:** Older adults, Kyphosis, Cobb angle, 6-minute walk test, Prescription medication, Body mass index, Hyperkyphosis, Upper extremity task, Physical function

## Abstract

Age-related hyperkyphosis is associated with adverse health outcomes, such as falls, fractures, and mortality. However, few studies investigated the relationship between the severity of hyperkyphosis and physical endurance in older adults. This study examined whether a degree of hyperkyphosis curvature was independently associated with the 6-minute walk test (6MWT) distance. We analyzed the baseline data of 112 older adults aged 60-92 enrolled in the Specialized Center of Research (SCOR) Kyphosis trial. The majority of the sample had at least a college degree and were white. On average, participants walked 503.9 (SD 82.3) meters in 6 minutes. Multivariate regression results showed that the degree of hyperkyphosis curvature was not independently associated with the 6MWT distance, but taller height, lighter weight, and less prescription medication were significant predictors of better performance on the 6MWT distance. Validation of the study findings in a large, diverse older adult population is warranted.

## Introduction

The population in the United States (U.S.) is aging rapidly. According to the U.S. Census Bureau, more than 54 million adults are ages 65 and older, and by 2050, the total number of adults ages 65 or older is projected to grow by approximately 85.7 million.^1^ Effective programs to reduce age-related risk factors are crucial to prevent or delay disease onset and slow the progression of illnesses. Promoting healthy aging and independent living has critical clinical and policy implications for increasing one's quality of life and reducing health care costs.

Age-related hyperkyphosis, excessive thoracic spine curvature, is one of the most common conditions among older adults.^2^ Kyphosis greater than 40° is defined as hyperkyphosis.^3,4^ Approximately 30% to 40% of older adults have hyperkyphosis,^5,6^ and its prevalence increases with age. In general, age-related hyperkyphosis is a potentially modifiable risk factor for adverse health outcomes, such as falls, fractures, pains, and mortality.^7^ The recent systematic review and meta-analysis reported that exercise interventions targeting hyperkyphosis might improve some health outcomes in adults with hyperkyphosis.^8^ Given a growing aging population in the U.S, the burden of age-related hyperkyphosis will become more significant. Thus, the development of more comprehensive knowledge and new insights into older adults with hyperkyphosis will assist in designing its prevention and treatment.^9^

Walking (gait) speed has been described as the sixth vital sign, given its ability to predict future health status,^10,11^ hospitalization,^12^ and mortality ^13^ among older adults. In general, walking speed decreases as one's age increases, although some older adults can retain gait speed despite aging. Additionally, the female sex, shorter height, greater body mass index (BMI), presence of chronic illnesses, and lifestyle (i.e., smoking, sedentariness) appear to be associated with slower walking speed.^14,15^ Moreover, a significant negative correlation has been reported between thoracic curvature angle and lung function restricting forced expiratory volume, forced vital capacity, quiet expiration intercostal thickness and deep expiration diaphragm muscle thickness.^16^ Thus, the severity of age-related hyperkyphosis may play an essential role in walking speed.

The 6-minute walk test (6MWT) is one of the most widely used assessment tools to measure aerobic capacity and endurance in older adults,^17,18^ and it has excellent validity and reliability.^18,19^ The 6MWT is affected by gait speed because slower gait speed reduces the distance walked during the timed test. The results of the 6MWT were associated with an increased risk of all-cause mortality in older adults.^20^ Furthermore, several systematic reviews examining factors associated with the 6MWT distance were conducted in adults with various health conditions (i.e., cardiovascular disease, chronic respiratory disease, acute respiratory distress syndrome), community-dwelling adults, and children/adolescents.^21-25^ However, the findings of these systematic reviews indicate that the 6MWT in older adults with hyperkyphosis is scarce, with only a little evidence showing significant negative effects of hyperkyphosis on the 6MWT.^26^ Thus, we investigated whether baseline Cobb's angle was independently associated with the 6MWT distance after controlling for potential confounding factors such as sociodemographic, extremity functions, and clinical characteristics in community-dwelling older adults with hyperkyphosis.

## Material and methods

### Design and sample

This is a cross-sectional, secondary data analysis of the Sex Differences in Musculoskeletal Conditions across the Lifespan Specialized Center of Research (SCOR) kyphosisrandomized controlled trial (RCT) in older adults. In this paper, we analyzed only the screening/baseline data of the SCOR kyphosis trial. A detailed description of the study design and eligibility of the study participants, and trial results have been published elsewhere.^27,28^ The study protocol was approved by the University of California, San Francisco, and Kaiser Permanente Northern California Institutional Review Boards.

In brief, eligibility criteria were as follows: proficient in English, age 60 years or older, kyphosis angle 40 degrees or higher (measured with a kyphometer at the screening visit), ability to walk one block without an assistive device, able to climb one flight of stairs independently, rise from a chair without the use of one's arms, no cognitive impairment on the Mini-Cog,^29^ and ability to pass safety tests in the screening examination or any disorder or disease likely to prevent or interfere with safe participation in a group-based exercise program, and ability to actively reduce their kyphosis measurement by at least 5 degrees. To assess the kyphosis eligibility criterion, we used the kyphometer tool (Techmedica Inc., Camarillo, CA) to obtain an external measurement of kyphosis (T3-T12) using a standard protocol. In the first measurement, participants were asked to stand in their usual posture, then a trained staff took a measurement. In the second measurement, they were instructed to have their best posture standing as straight and tall as they could. If participants had a difference between the two measures equal to or greater than 5 degrees (flexible spine), they were included in the study. If the difference between the two measures were less than 5 degrees, we defined it as a fixed spine. Those participants were excluded from the study. (Note: This paper analzyed the baseline data of the SCOR kyphosis trial. However, participants needed to have a flexible spine because the SCOR kyphosis trial included the exercise and posture intervention to improve hyperkyphosis).

We recruited participants from local senior centers and outpatient clinics at 2 large urban medical centers in San Francisco, California. We pre-screened 305 adults by telephone or online and of those, 95 did not meet eligibility criteria or were no longer interested in participating in the study. 210 were invited for a further screening baseline visit, but 90 did not meet all eligibility criteria (e.g., did not pass kyphosis measurement, had a fixed spine, failed Mini-Cog, failed safety exam). Of the remaining 120 participants who met all eligibility criteria, 8 declined to continue to be in the study. A total of 112 participants were analyzed in the current study. Written informed consent was obtained from all participants before study procedures. For participants' safety, permission from the potential participant's primary care provider was obtained before randomization.

### Baseline measures

**6MWT** measured the distance in meters covered while walking on a flat, hard surface (i.e., a long hallway) for 6 minutes.^30^ It measures the global and integrated responses of systems involved during exercise and has been suggested as a valid test reflective of daily living activities.^31^ **Cobb angle of kyphosis** was measured using the gold standard Cobb angle of kyphosis derived from standing lateral spine radiographs and a standardized protocol for thoracic kyphosis (T4-T12).^32^ In brief, participants stood barefoot with knees straight and arms supported at 90° of flexion. They were instructed to hold full inhalation for the duration of the scan. Measurements were made by a trained radiologist. A greater Cobb angle indicates more kyphosis severity.

**Weight, height, and BMI** were measured with usual standing height in centimeters and barefoot weight in kilograms with light clothing using standard methods and calculated BMI as weight in kilograms divided by height in meters square. **Vertebral fractures** were calculated from T4–L4 baseline standing lateral spine radiographs using the Genant semi-quantitative (SQ) method grading fractures ranging from 0 = none (normal), 1 = mild, 2 = moderate, and 3 = severe.^33^ In the current study, we defined vertebral fracture as S. Q. ≥ 1. **Upper extremity function** was measured using three activity of daily living (ADL) extremity tests (putting on and removing a laboratory coat, picking up a penny from the floor, and lifting a 7-lb. book to a shelf) from the modified Physical Performance Test (modified PPT).^34,35^ **Average baseline steps per day** were measured using an Omron pedometer to objectively calculate the baseline physical activity level for 7 consecutive days before the randomization visit. **Sociodemographics** such as age, sex, race/ethnicity, and education were collected from participants at the baseline visit. **Medication** and supplement information was obtained by asking participants to self-report the names of any medications/supplements they regularly had taken to manage their health and comorbidities. We classified them into two main categories (prescription, over-the-counter, and supplements). The prescription medications were further categorized into disease conditions (i.e., blood pressure, hyperlipidemia, hypothyroidism, diabetes, anxiety/depression, arthritis, and osteoporosis).

### Statistical analysis

Descriptive statistics were used to describe participants’ sociodemographic (age, gender, race, education) and clinical information (height, weight, BMI, 6MWT, Cobb’s angle, vertebral fractures, daily baseline steps, 3 extremity ADL tasks, number and types of medication). To investigate the association between sociodemographic and clinical factors on participants’ performance on the 6MWT distance (meter), univariate and multivariate regression analyses were conducted. The multivariate regression model tested whether baseline Cobb’s angle was independently associated with the 6MWT performance after controlling for potential confounding factors such as sociodemographic, extremity functions, and clinical characteristics. Statistical significance was set at *P* < .05. All analyses were conducted using SPSS (version 21.0; IBM, Chicago, IL, USA).

## Results

### Sample characteristics

Table 1 shows the sample sociodemographic and clinical characteristics. Of the 112 participants, the mean [standard deviation (S.D.)] age was 69.8 (± 6.3) years with a range from 60 to 92 years, 67 (59.8%) were women, 102 (91.1%) were white, 98 (87.5%) had a bachelor's or advanced degree. Mean (SD) height (cm), weight (kg), and BMI (kg/m^2^) were 167.0 (± 9.5) cm, 73.9 (± 14.9) kg, and 26.4 (± 4.0) kg/m^2^, respectively. The mean (S.D.) number of prescription medications and supplements and the number of prescription medications alone was 5.8 (3.6) and 2.9 (2.2), respectively. Mean (S.D.) Cobb's angle was 55.6 (± 12.1) degrees. 19 (17.0%) participants had at least one vertebral fracture. The average (S.D.) baseline steps per day were 6226.1 (± 3363.8). For the three ADL tasks (i.e., book lift, jacket, pick up penny tasks), the average (S.D.) seconds taken to complete the task was 2.5 (± 0.7) seconds for the book lift task, 9.8 (± 3.4) seconds for jacket task, and 1.6 (± 0.6) seconds for pick up penny task. On average (S.D.), participants walked 503.9 (± 82.3) meters in the 6MWT and 6226 (± 3363.8) steps per day.

### Regression analyses

Table 2 presents the results of univariate regression analyses and a multivariate linear regression analysis predicting individuals' performance on the 6MWT. Overall, the multivariate regression model was significant (adjusted R^2^ = .394, *p* < .001). Results revealed that Cobb’s angle was not significantly associated with 6MWT distance (*β* = −0.52; 95% CI, −1.68 to 0.64; *p* = .378) even after controlling for potential confounding factors However, the model identified 3 significant predictors: (1) height (cm) (*β* = 4.18; 95% CI, 1.79 to 6.56; *p* = .001), (2) weight (kg) (*β* = −2.73; 95% CI, −3.97 to −1.50; *p* < .001), and (3) number of prescription medications taken (*β*= −11.20; 95% CI, −17.54 to −4.87; *p* = .001). That is, participants with taller height, lighter weight, and took less prescribed medication were significantly more likely to perform better in the 6MWT than their counterparts.

## Discussion

Age-related hyperkyphosis has been under-investigated due to the lack of standardized diagnostic criteria and treatments. This paper aimed to examine whether Cobb angle of kyphosis derived from standing lateral spine radiographs was significantly associated with the 6MWT distance controlling for potential confounding factors. While we hypothesized that the study participants with a greater degree of Cobb angle of kyphosis would have a significantly shorter 6MWT distance, we did not find its significant association in community-dwelling older adults enrolled in the SCOR kyphosis trial. This non-significant association finding agrees with the previous study result.^9,36,37^ We consider several possible explanations for the non-significant association between 6MWT distance and degree of kyphosis. First, age-related hyperkyphosis progresses slowly (approximately 3 degrees each decade of life)^38^ in adults over 50 years of age. Thus, the older participants in the present study might have time to adapt to slow changes in their bodies and maintain exercise endurance.^9^ Second, the study sample represented relatively physically active community-dwelling older adults. For example, this study sample's mean daily step counts were 6226.1 (SD 3363.8), higher than the national representative adults in the U.S.^39,40^ Third, we excluded the older adults with a fixed kyphosis or those without at least 5 degrees of mobility in the thoracic spine from the study because they were less likely to respond to the SCOR kyphosis exercise and posture intervention.^16^ It is possible that those older adults could have reduced pulmonary function from age-related hyperkyphosis, and they might have a significantly shorter 6MWT distance.^16^

Consistent with the findings from the systematic review,^24^ taller heights and lighter weights were significant predictors for better 6MWT distance. This is because older adults with taller heights had longer walking stride lengths, and those with lighter weight had faster-walking speeds than their counterparts. Other studies have examined BMI, instead of height and weight, and shown that greater BMI was significantly associated with shorter 6MWT distance.^41^ Age is also considered one of the most significant factors in predicting the 6MWT distance. In general, as age increases, the 6MWT distance becomes shorter. However, age becomes no longer significant after controlling for other factors in this study. One potential explanation is the homogeneity of the older adult sample in this study, selecting community-dwelling older adults with hyperkyphosis using tight RCT eligibility criteria.

In this study, the participants reported using fewer prescription medications (mean 2.9 per day) than what might be considered average for their age group. U.S. older adults who took 5 or more medications increased from 12.8% to 39.0% between 1988-1991 and 2009-2010, respectively.^42^ Despite the relatively fewer prescription medication intake, participants who took more prescription medications had significantly shorter 6MWT distances than those who took less in this study. This finding is intuitive and aligns with the previous study finding in older adults with hyperkyphosis.^9^ Older adults taking more prescription medications might have more chronic illnesses and worsening health status. In this study, a small number of participants who took arthritis medication were not significantly impacted by joint pain from osteoarthritis.

### Strengths and limitations

To the best of our knowledge, this study is one of few investigations to examine the association between the 6MWT and Cobb angle of kyphosis in older adults with age-related hyperkyphosis. Another strength includes using the gold-standard Cobb angle measurements of kyphosis derived from standing lateral spine radiographs, a costly and time-consuming measure. Despite these strengths of this study, several limitations need to be addressed. Older adults with fixed hyperkyphosis or multiple comorbidities prohibiting participation in a posture training and exercise program were excluded from the study. Therefore our results may reflect a healthier group of hyperkyphotic subjects. In addition, the study subjects were primarily white, highly motivated, educated with at least a college education, and physically active. Thus, the findings of this study may not be generalizable to all older and ethnically diverse adults. Lastly, due to cross-sectional analysis, causal relationships cannot be inferred, or there might be unknown confounding factors that we did not adjust for in this study.

## Conclusions

This study found that the degree of hyperkyphosis curvature was not independently associated with the 6MWT distance in older adults even after controlling for known confounding factors. In contrast, the older adults who were tall, light in weight, and taking fewer prescription medications had a greater 6MWT distance. This study highlights the need for considering prescription medications when estimating height and weight-adjusted 6MWT distance in older adults with hyperkyphosis. Validation of the study findings in a large, diverse older adult population is warranted.

## Figures and Tables

**Table 1:** Baseline sample sociodemographic and clinical data (N = 112).

Sociodemographics		Mean (SD) or %(n) [Range]
Age (years)	-	69.8 (6.3) [60-92]
Gender	Men	40.2 (45)
	Women	59.8 (67)
Race/Ethnicity	White	91.1 (102)
	Non-white	8.9 (10)
Education	Completed high school or some college education or less than high school	12.5 (14)
	Completed college or graduate school	87.5 (98)
**Clinical data**		
Height (cm)	-	167.0 (9.5) [146.8-189.0]
Weight (kg)	-	73.9 (14.9) [44.1-117.4]
Body Mass Index (kg/m^2^)	-	26.4 (4.0) [19.1-37.1]
Number of prescriptions or over the counter medication, or supplement	-	5.8 (3.6) [0-15]
Number of prescription medication	-	2.9 (2.2) [0-9]
Taking blood pressure medication	Yes	38.4 (43)
Taking hyperlipidemia medication	Yes	25.0 (28)
Taking hypothyroidism medication	Yes	16.1 (18)
Taking diabetes medication	Yes	2.7 (3)
Taking anxiety/depression medication	Yes	19.7 (22)
Taking arthritis medication	Yes	3.6 (4)
Taking medication(s) for osteoporosis Alendronate. Other antiresorptive medication, parathyroid hormone, or other bone-building medications	Yes	9.8 (11)
6-Minute Walk Test (meters)^a^	-	503.9 (82.3) [270.8-722.2]
Kyphosis by Cobb's angle (**°)**^b^	-	55.6 (12.1) [28.0-83.7]
Vertebral fractures ^c^	Yes (1 or more)	17.0 (19)
Average baseline steps (per day)^d^	-	6226.1 (3363.8) [757.9-21931.0]
Book lift task (seconds)	Yes / seconds	2.5 (0.7) [1.3-6.0]
	No	0
Jacket task (seconds)	Yes / seconds	9.8 (3.4) [4.6-26.9]
	No	0
Pick up penny task (seconds)^e^	Yes / seconds	1.6 (0.6) [0.7-4.2]
	No	0

a = 2 missing cases

b = 3 missing cases

c 1 participant had 4 fractures, 2 had 3 fractures, 5 had 2 fractures, and 11 had 1 fracture

d = 18 missing cases

e = 1 missing case

**Table 2:** Unadjusted and adjusted linear regression models predicting the 6-minute walk test distatnce at baseline (N = 106).

	Unadjusted				Adjusted^a^			
	*β*	t	p-value	95% CI	*β*	T	p-value	95% CI
Age	−3.81	−3.17	.002	−6.19 to −1.43	−2.05	−1.86	.066	−4.23 to 0.14
Female	−8.87	−0.55	.582	−40.72 to 22.97	−10.22	−0.44	.661	−56.31 to 35.86
Non-white	−62.33	−2.33	.022	−115.36 to −9.30	−34.55	−1.50	.136	−80.20 to 11.11
Completed college or graduate school	8.55	0.36	.718	−38.29 to 55.40	23.66	1.24	.217	−14.16 to 61.48
Height (cm)	2.30	2.90	.005	0.73 to 3.88	4.18	3.47	.001	1.79 to 6.56
Weight (kg)	−0.58	−1.10	.276	−1.63 to 0.47	−2.73	−4.39	<.001	−3.97 to −1.50
Vertebral fractures	7.67	0.36	.719	−33.05 to 53.70	24.84	1.28	.203	−13.61 to 63.29
Cobb’s angle (°)	0.36	0.55	.583	−0.95 to 1.67	−0.52	−0.89	.378	−1.68 to 0.64
Book lift seconds	−24.31	−2.37	.020	−44.68 to −3.94	−1.42	−0.14	.889	−21.61 to 18.76
Jacket seconds	−7.32	−3.31	.001	−11.70 to −2.94	−4.99	−1.97	.052	−10.02 to 0.05
Penny seconds	−39.17	−3.12	.002	−64.04 to −14.30	−8.98	−0.63	.533	−37.47 to 19.51
Number of prescription medication	−14.05	−4.21	<.001	−20.66 to −7.43	−11.20	−3.51	.001	−17.54 to −4.87

a N = 106; Model R^2^ = .462, Adjusted R^2^ = .394, *p* < .001
